# Design of Customize Interbody Fusion Cages of Ti64ELI with Gradient Porosity by Selective Laser Melting Process

**DOI:** 10.3390/mi12030307

**Published:** 2021-03-15

**Authors:** Cheng-Tang Pan, Che-Hsin Lin, Ya-Kang Huang, Jason S. C. Jang, Hsuan-Kai Lin, Che-Nan Kuo, De-Yao Lin, Jacob C. Huang

**Affiliations:** 1 Department of Mechanical and Electro-Mechanical Engineering, National Sun Yat-Sen University, Kaohsiung City 80424, Taiwan; chehsin@mail.nsysu.edu.tw (C.-H.L.); ken168k@mem.nsysu.edu.tw (Y.-K.H.); 2 Institute of Precision Medicine, National Sun Yat-Sen University, Kaohsiung City 80424, Taiwan; 3 Department of Mechanical Engineering, National Central University, Taoyuan City 32001, Taiwan; jscjang@ncu.edu.tw; 4 Institute of Materials Science and Engineering, National Central University, Taoyuan 32001, Taiwan; 5 Graduate Institute of Materials Engineering, National Pingtung University of Science and Technology, Pingtung City 91201, Taiwan; HKLin@mail.npust.edu.tw; 6 Department of Bioinformatics and Medical Engineering, Asia University, Taichung City 41354, Taiwan; cnkuo@asia.edu.tw; 7 Industrial Technology Research Institute, Hsinchu 31040, Taiwan; dylin@itri.org.tw; 8 Hong Kong Institute for Advanced Study, Department of Materials Science and Engineering, City University of Hong Kong, Kowloon, Hong Kong; chihuang@cityu.edu.hk

**Keywords:** cage, selective laser melting, 3D printing, Ti64ELI, stress concentration, stress shielding, gradient porosity

## Abstract

Intervertebral fusion surgery for spinal trauma, degeneration, and deformity correction is a major vertebral reconstruction operation. For most cages, the stiffness of the cage is high enough to cause stress concentration, leading to a stress shielding effect between the vertebral bones and the cages. The stress shielding effect affects the outcome after the reconstruction surgery, easily causing damage and leading to a higher risk of reoperation. A porous structure for the spinal fusion cage can effectively reduce the stiffness to obtain more comparative strength for the surrounding tissue. In this study, an intervertebral cage with a porous gradation structure was designed for Ti64ELI alloy powders bonded by the selective laser melting (SLM) process. The medical imaging software InVesalius and 3D surface reconstruction software Geomagic Studio 12 (Raindrop Geomagic Inc., Morrisville, NC, USA) were utilized to establish the vertebra model, and ANSYS Workbench 16 (Ansys Inc., Canonsburg, PA, USA) simulation software was used to simulate the stress and strain of the motions including vertical body-weighted compression, flexion, extension, lateral bending, and rotation. The intervertebral cage with a hollow cylinder had porosity values of 80–70–60–70–80% (from center to both top side and bottom side) and had porosity values of 60–70–80 (from outside to inside). In addition, according to the contact areas between the vertebras and cages, the shape of the cages can be custom-designed. The cages underwent fatigue tests by following ASTM F2077-17. Then, mechanical property simulations of the cages were conducted for a comparison with the commercially available cages from three companies: Zimmer (Zimmer Biomet Holdings, Inc., Warsaw, IN, USA), Ulrich (Germany), and B. Braun (Germany). The results show that the stress and strain distribution of the cages are consistent with the ones of human bone, and show a uniform stress distribution, which can reduce stress concentration.

## 1. Introduction

**Figure 1:**
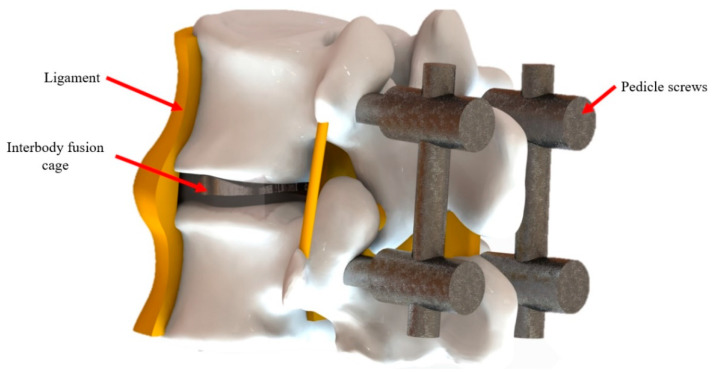
The schematic details of spinal fusion surgery.

With the advance in technology and innovation, the changes in human life has led to an increase in the risk of waist injuries in activities as a result of traumatic injury of the waist due to intense exercise or prolonged posture problems during youth. Spinal trauma is often accompanied by many kinds of fracture or dislocation that causes instability of the spine and impaired nerve function. Many diseases are often caused by aging in later life. In addition, non-damage factors such as a tumor or intervertebral disc lesions that press the spinal nerves cause pain in the back. In the early 1980s, Harms and Rohlinger presented transforaminal interbody fusion [1], which has become a widely accepted procedure for patients with degenerative spine diseases such as vertebral trauma, spondylolisthesis, and disc degeneration [2,3,4,5,6,7,8]. Spinal fusion is an orthopedic surgical technique that joins two or more vertebrae. It can be used to treat a variety of conditions affecting any level of the spine (lumbar, cervical, and thoracic). Additional hardware such as screws, plates, and cages are often used to secure the bones in place when the graft fuses the two vertebrae together, as schematically shown in Figure 1. Successful vertebral fusion surgery mainly depends on the postoperative recovery, which is related to the patient’s bone density, cage placed position, and the structure design. The cage placement position is closely related to its own design of the mechanical structure. In recent years, implant-related technologies such as artificial implants and cages have rapidly developed, and more options for spinal reconstruction surgery have been provided. With the development of spinal implants, the most successful artificial replacement implant has been the cage [9,10,11,12,13,14]. 

Various materials for intervertebral cages have been introduced and gradually applied in clinical experiments. Bagby and Kuslich were the first to use interbody fusion cages (BAK cage) for lumbar interbody fusion surgery in 1988 [14]. It had a high chance of success to maintain the height of the upper and lower vertebral bodies and could also maintain the width of the nerve hole between the anterior and arcus vertebra to reduce the requirement of using a fixator. The studies showed that various risk factors that affect cage retropulsion after lumbar interbody fusion (LIF) have been reported [15,16,17]. LIF is a spine fusion specific to the lumbar region. Previous studies have suggested that additional posterior instrumentation is critical for preventing cage retropulsion, particularly in terms of flexion–extension torque. Therefore, various LIFs were recommended for fusion surgery for different portions of bones [18,19]. Uzi et al. [20] reported that cage retropulsion could occur during flexion movement and thus suggested that this could be prevented by additional posterior instrumentation. However, there are still many problems with the acceleration of deterioration in the adjacent parts of the intervertebral disc. The main reason is the difference in mechanical properties between the artificial implant and the surrounding human tissue.

Recently, additive manufacturing (AM), or rapid prototyping or 3D printing, to build materials directly into the final 3D shape, has become popular. The end products can be made by depositing materials layer by layer; each layer is designed with original data by computer aided design (CAD) [21]. Selective laser melting (SLM) has been developed successfully for metal powders and the density of the solid part fabricated by SLM can be higher than 99% [22]. Among all pure metals and metallic alloys applied for the biomedical implant, commercial pure (CP) Ti and Ti-based alloys remain the better choices for hard tissue replacement due to their excellent mechanical, physical, and biological performance [23]. In comparison with the elastic moduli to other biocompatible alloys for implants such as the Co–Cr alloy (210–253 GPa) and 316L stainless steel (190–210 GPa), the modulus of the Ti-based alloys is lower (100–140 GPa) [24], but is still much higher than the Young’s modulus of the human porous tissues they replace (4–30 GPa) [25]. The higher Young’s modulus of implants could make the implant basically sustain the load alone, which leads to the decreased loading on a bone, and the bone would become less dense and weaker because there is no stimulus hat is required to maintain bone mass. This unwanted phenomenon, the so-called stress shielding effect, leads to bone osteoporosis [26]. In order to further reduce and match the modulus of the Ti-based parts with human bones, applying porous structures could be a solution. There have been some natural porous materials such as zeolites, activated carbon, and spongy bone. Porous materials are well known to possess unique characteristics such as low density, high-surface–area ratio, and high mechanical energy absorption efficiency [27]. In order to promote human tissues to grow into the pores, the desired micro-pore size in the porous implant metals needs to be controlled at the scale about 300 to 800 μm, according to previous studies and review papers [28,29,30,31,32,33]. Some previous studies have demonstrated that the nanostructured surface morphology of the implant is an important property for osseointegration [34,35,36,37]. Nano-structured surface morphology is favorable for cell attaching on the surface, but not really for whole bone-cell ingrowth [38]. The normal bone cells, typically several micrometers in size, would be much larger than the nanostructured pores [39]. 

In this study, the open-cell pore sizes were designed in the micrometer range, which is beneficial to whole bone-cell ingrowth. Usually, pore sizes more than 300 μm are recommended due to the enhanced new bone formation and surrounding tissue. Due to vascularization, pore size has been shown to affect the progression of osteogenesis. Small pores would favor hypoxic conditions and would induce osteochondral formation before osteogenesis while large pores that are well-vascularized would lead to direct osteogenesis without preceding cartilage formation. In some previous efforts, porous metallic foams have been fabricated by powder metallurgy by mixing metallic and salt powders [40,41,42]. In this study, SLM was adapted to prepare similar metallic porous structures, and many kinds of porous structures are discussed. The resulting microstructure and mechanical properties are also reported.

## 2. Experimental Methods

### 2.1. Porous Cage Design 

**Figure 2:**
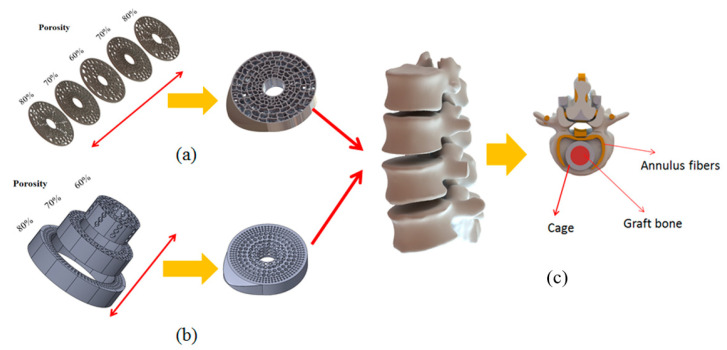
Gradient porosity structure for cages. (**a**) Vertically gradient porosity. (**b**) Horizontally gradient porosity. (**c**) Cage placement in spinal fusion surgery.

In this study, the SolidWorks computer-aided design software program (SolidWorks 2016, Dassault Systems Inc., Velizy, France) was used to create cylindrical models with gradient porosity with outside diameters of 20 mm, pore sizes of 800 and 3500 μm, and thicknesses of 10 mm. However, for human implants, spongy structures with gradient porosity are essential to avoid the stress concentration effect. Therefore, three different kinds of porosities were developed for cages. To obtain gradient porosity effects, the three different kinds of porosities were integrated into one fusion cage. As shown in Figure 2a, the porosities from top to bottom were determined to be 80–70–60–70–80% with the outer diameter of this model was 20 mm, each layer’s thickness was 2 mm, and the center was a hollow cylinder. 

Figure 2b, from outside to inside, was determined as 80%, 70%, and 60% with the outer diameter of 20 mm, the second ring diameter of 15 mm, the third ring diameter of 10 mm, and the thickness of 10 mm; the inner part was a hollow cylinder. These two kinds of gradient directions were compared to find out which one was better. And using the design to perform the spinal fusion surgery, as shown in Figure 2c.

### 2.2. Additive Manufacturing with Selective Laser Melting (SLM)

**Figure 3:**
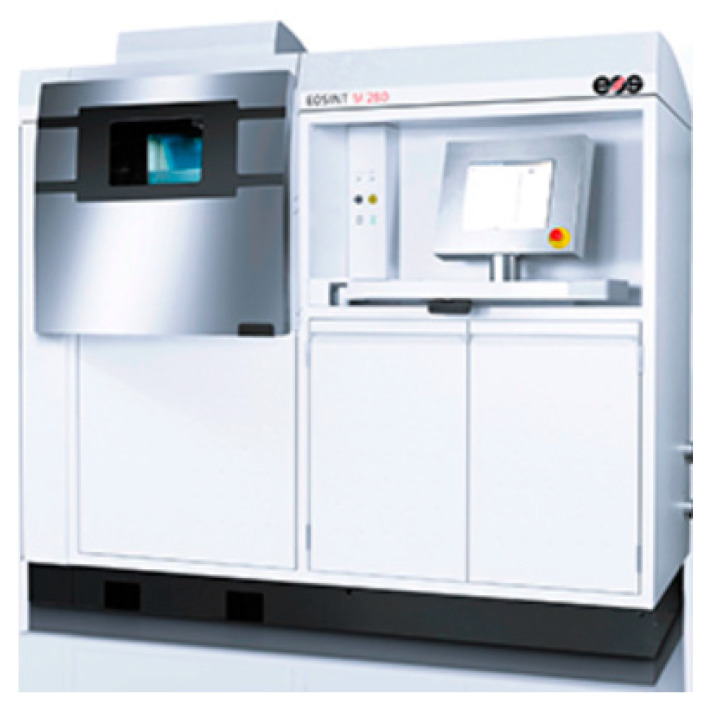
The EOSINT M 280 selective laser melting (SLM) system.

In this study, a fully automatic EOSINT M 280 was used to fabricate test samples directly from three-dimensional CAD design data. The machine is shown in Figure 3. It produces components by means of AM without any other tools. Open-cell porous sample models were designed by using the SolidWorks CAD software (SolidWorks 2016, Dassault Systems Inc., Velizy, France ), and made with different porosities and random micropore sizes ranging from 800 to 1500 μm. The structures of the SLM-processed fusion cages were examined by SEM (Scanning Electron Microscope) and OM (Optical Microscope). The metal powder material used in this study was titanium Ti64ELI, which is a kind of titanium alloy for EOSINT M systems (Electro-Optical Systems Inc., Krailling, Germany). The sample was formed by using the melting source of the laser beam and welding layer by layer. Finally, the porous samples were cleaned to reduce residual incomplete melted powders and separated from the substrate by wire electrical discharge machining.

### 2.3. Stress-Relief Annealing

Ti64ELI is widely used among the titanium alloys, and usually in an annealed state. This study executed stress-relief annealing treatment on the cages were made by the SLM process, in order to eliminate the residual stress after processing of the titanium alloy. The UF-E3FS (Ultra Fine Technology Inc., Taoyuan, Taiwan) high-temperature furnace was used in this experiment. In the experimental method, the stress-relief annealing condition was set as ranging from 450 to 650 ℃ with an annealing time of 40, 80, 120, 160, 200, and 240 min, respectively. The longest duration was up to four hours. Additionally in additive manufactured metallics, anisotropy in microstructure and mechanical property is also observed [43]. Furthermore, the stress-relief annealing could reduce the residual stress, but also regulate the phase composition and mechanical properties [44,45]. The parametric conditions of the stress relief annealing experiments in this article were based on the technical report of “Heat treatment of titanium and titanium alloys” provided by NASA Technical Reports Server (NTRS). The best stress relief annealing condition of material Ti64ELI was set from 537 to 648 °C and 15 to 60 min in the report. The authors enlarged the interval and utilized uniform design experimentation to uncover the mechanical properties and the optimal parameters.

Uniform design experimentation (UDE) was used to determine the best parameters of the annealing process. The uniform design only considers the parameters of the test group to be evenly distributed in the overall experiment. This means that the representative groups in the experiment are selected according to the principle of “uniform dispersion”, which can ensure that the test points have evenly distributed statistical characteristics so that each sample needs to be tested only once. It focuses on the uniform spread of experimental parameters in the scope of the test in order to gain the most information with the least amount of experiment.

### 2.4. Mechanical Test of Porous Structure

**Figure 4:**
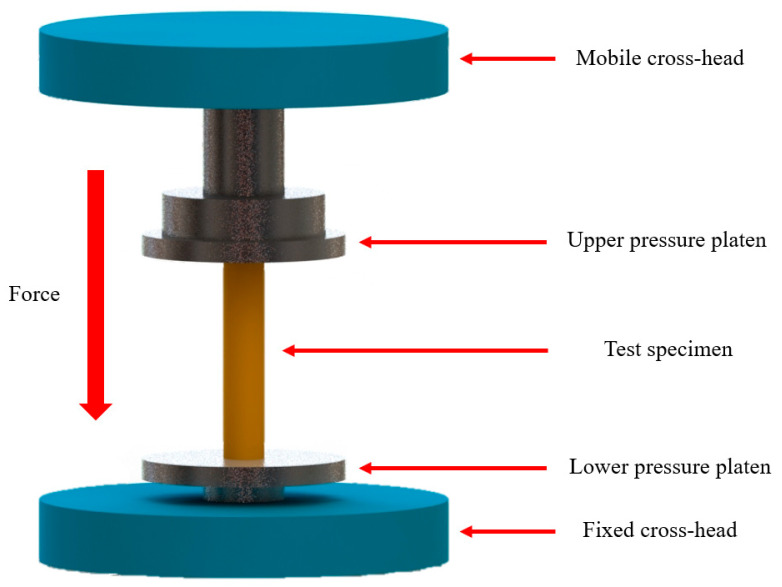
The compression test of the universal testing machine.

**Figure 5:**
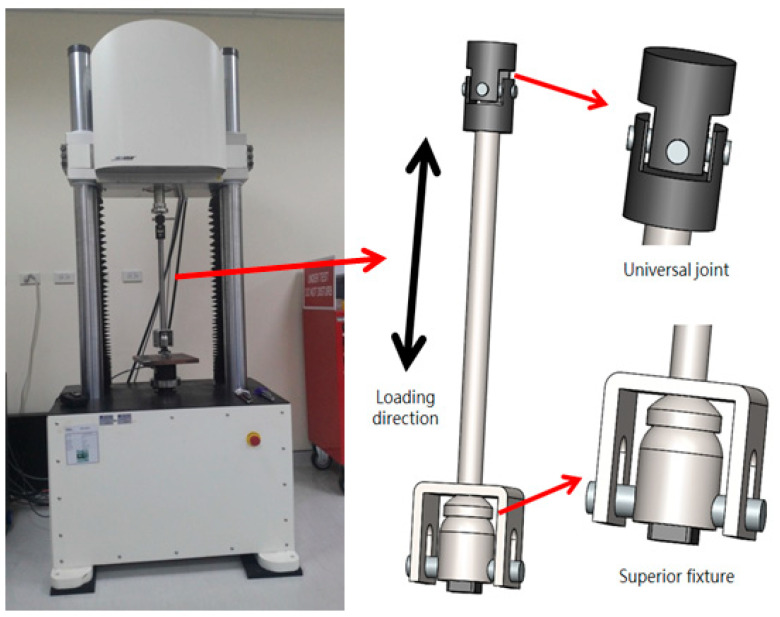
Instron 5582 universal testing machine and SHIMADZU HMV-2 Vickers microhardness test machine.

In this study, the Ti64ELI alloys of gradient porosity of cages made by the SLM process were tested using compression. Sandpaper was used to grind the top and bottom of samples before the experiment, which can ensure that the samples and the platens have a flatter contact surface during the compression test. The compression test of a universal testing machine is shown in Figure 4. The samples were tested under compression with a strain rate of 1 × 10^−4^ s^−1^ at room temperature by using the Instron 5582 (Instron Inc., Norwood, MA, USA) universal testing machine equipped with a 100 kN load cell and Instron 2601 Linear Variable Differential Transformer (LVDT) displacement transducer, as shown in Figure 5.

The microhardness test machine used was a SHIMADZU HMV-2 (Shimadzu Corporation, Kyoto, Japan) Vickers test, as shown in Figure 5. Before the test, the samples with the gradient porosity were ground and polished by using silicon carbide sandpaper numbers 800, 1000, 1500, and 2000 with water. For this test, a 4.903 N load was applied for 15 seconds. Each sample was taken at nine test points and the average of these values was the hardness.

**Figure 6:**
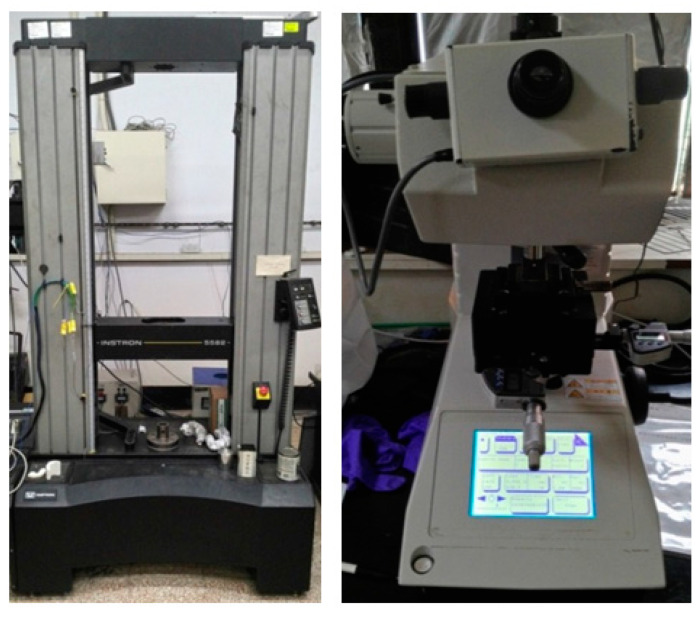
Dynamic testing machine Bose 3510.

According to the legislation of the Ministry of Health and Welfare of Medical Equipment, the fatigue test is one of the performance tests. The related regulations of the method are based on the American Society for Testing and Materials (ASTM) F2077-17. It describes the terminology, specification, method, and procedure for dynamic tests. In this study, three samples were applied with a well-defined axial force-cycle of 25% of yielding stress (2020 N) to 10% of maximum load (200 N), and the frequency was 5 Hz for about 1.5 million times. The testing machine was a Bose 3510, as shown in Figure 6, with the contact part of polyoxymethylene (POM) to simulate bone. After the test, samples were observed to identify if there were any cracks or deformation.

### 2.5. Simulation

The lumbar spine model constructed in this study was assumed to be homogenous and isotropic. The complete lumbar spine model (L1–L4) was used as the control group. The intervertebral disc between L2 and L3 was removed, and replaced by the interbody cage as a test group. As the direct load on L1 and L4 was limited by the boundary conditions, the actual stress distribution of the vertebral body will not be a true situation. Therefore, the intervertebral disc between the L2 and L3 was removed and replaced by the fusion cage and supplemented with bilateral pedicle screw fixation. The bottom of the L4 was fixed in all directions. The compressive load of 280 N and the moment of 7.5 Nm were applied to the upper surface of L2 as described in previous literature [46,47,48,49]. The compressive load of 280 N corresponds to the partial weight of a human body, and the moment of 7.5 Nm simulates the motion modes occurring in different conditions such as flexion, extension, lateral bending, and axial rotation. Considering the symmetry of the sagittal plane, this study simulated the biomechanical properties of surgical finite element models with vertical compression and the four motion modes: flexion, extension, lateral bending, and axial rotation. The mechanical data were expressed using von Mises stress contours. The material properties are shown in Table 1.

## 3. Results and Discussions

### 3.1. Gradient Porous Fusion Cage Dimensions

**Figure 7:**
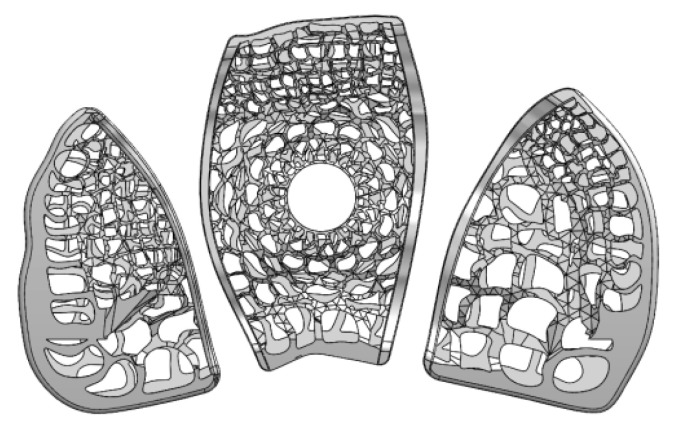
Cage design was separated into three parts.

**Figure 8:**
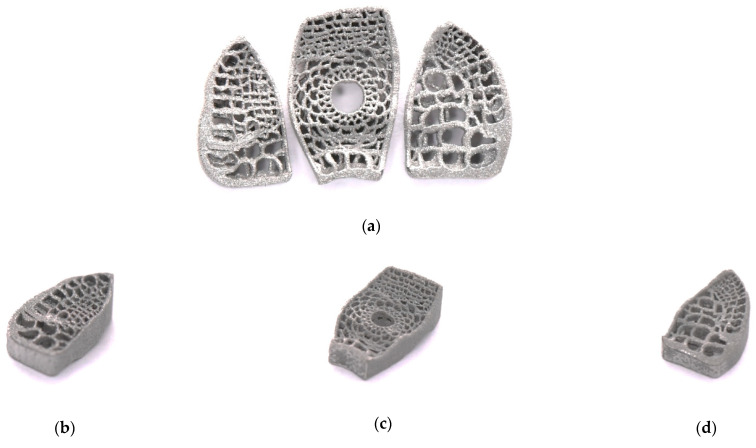
Fusion cages fabricated with SLM. (**a**) Three parts of design, (**b**) Left, (**c**) middle, (**d**) right.

**Figure 9:**
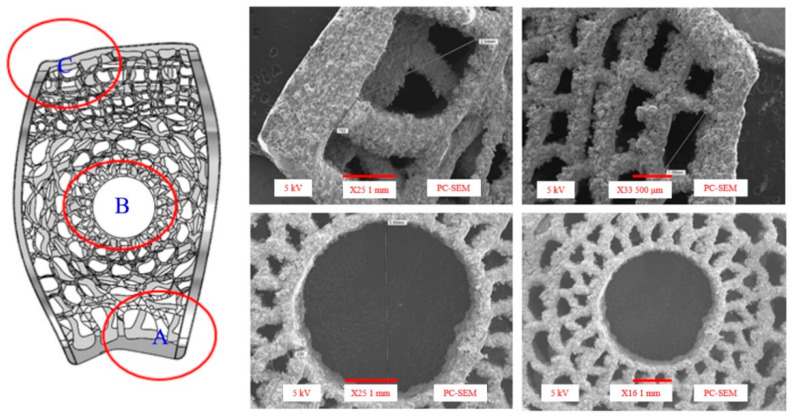
The scanning electron microscope (SEM) photograph for specimens.

**Figure 10:**
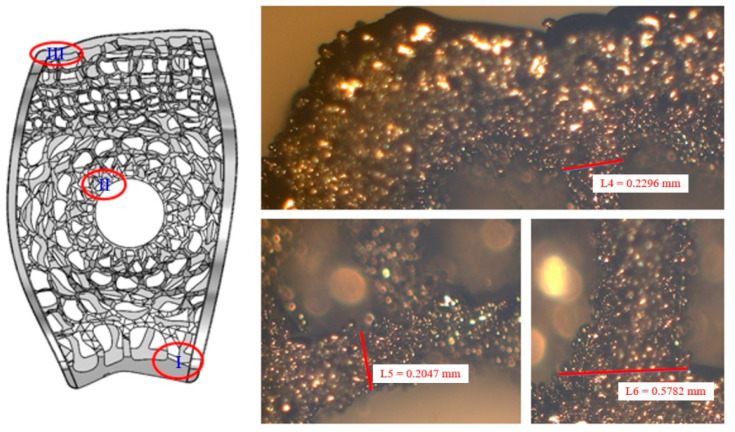
The optical microscope (OM) photograph for specimens.

Figure 7 and Figure 8 show samples of fusion cages with gradient porosity fabricated by SLM. There were a total of six groups of products. The design was separated into three parts which were left (b), middle (c), and right (d) for the mechanical tests. Many semi-melted powders had accumulated on the pore area and surfaces, causing the pores to be blocked and stuffed with the powders. This is because the pores are so small that powder cannot be blown away by an air gun and accumulate during the printing process. Therefore, to some extent, enlarging the pore model can effectively reduce the phenomena of powder accumulation. The as-printed fusion cages showed consistent geometric agreement with the design models, as shown in Figure 9 and Figure 10. The average widths were 9.44 mm, 11.2 mm, and 11.7 with standard deviations of 0.25, 0.43, and 0.20, which were close to the design of 9.5 mm, 11.3 mm, and 11.8 mm, respectively. The average heights were 5.99 mm, 6.08 mm, and 6.06 with standard deviations of 0.25, 0.25, and 0.03, which were close to the design of 6 mm, 6 mm, and 6 mm, respectively. The average depths were 15.93 mm, 16.42 mm, and 18.18 with standard deviations of 0.33, 0.33, and 0.43, which were close to the design of 16 mm, 16.5 mm, and 18.4 mm, respectively. The results show that real porosity would be lower than the designed porosity, and dimensions measured by optical microscope (OM) and SEM are shown in Table 2, Table 3, Table 4, Table 5, Table 6 and Table 7. The average beam dimensions measured by SEM were 0.277 mm, 0.229 mm, and 0.655 mm with standard deviations of 0.25, 0.20, and 0.64, which were close to the design of 0.25 mm, 0.20 mm, and 0.64 mm, respectively. The average hole dimensions measured by SEM were 1.063 mm, 3.373 mm, and 2.188 mm with standard deviations of 0.018, 0.113, and 0.173, which were close to the design of 1.22 mm, 3.54 mm, and 2.34 mm, respectively. The average beam dimensions measured by OM were 0.2317 mm, 0.2328 mm, and 0.4926 mm with standard deviations of 0.028, 0.0169, and 0.0458, which were close to the design of 0.12 mm, 0.21 mm, and 0.59 mm, respectively. The design of the interbody fusion cage compared with the actual products made by SLM was less than 2%. On the tiny structure (bone beam, hole), the actual size of the bone beam was larger than the design size due to the marginal effect of the laser beam diffusion. The external size was small and the error was about <15%. The hole was less affected, and the error was about <10%. The results suggest that various fusion cage model designs can be printed using SLM, as seen in Table 1, Table 2, Table 3, Table 4, Table 5, Table 6 and Table 7.

### 3.2. Annealing and Microhardness Analysis

**Figure 11:**
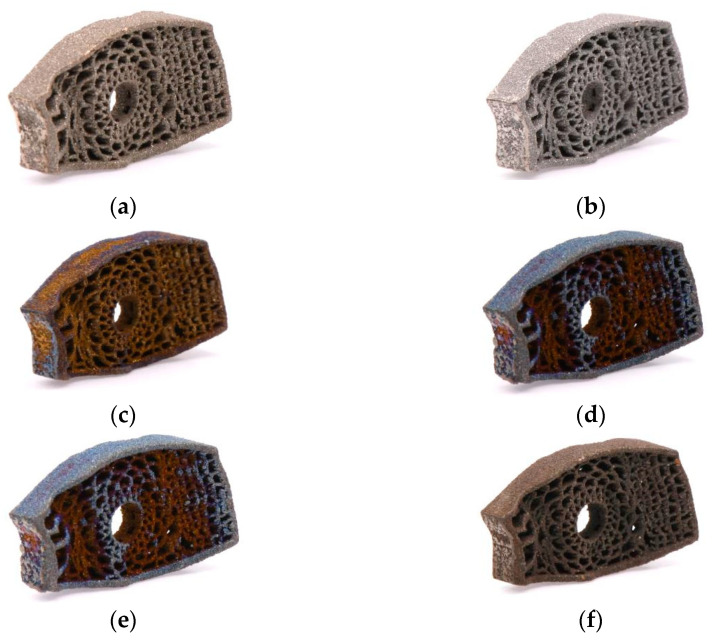
Annealing parameters (**a**) 450 °C/120 min; (**b**) 490 °C/240 min; (**c**) 530 °C/80 min; (**d**) 570 °C/200 min; (**e**) 610 °C/40 min; (**f**) 650 °C/160 min.

**Figure 12:**
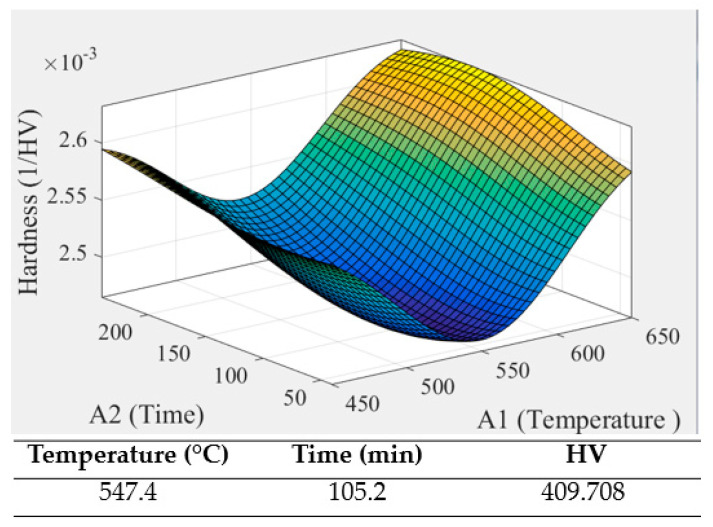
The optimal parameter for annealing.

**Figure 13:**
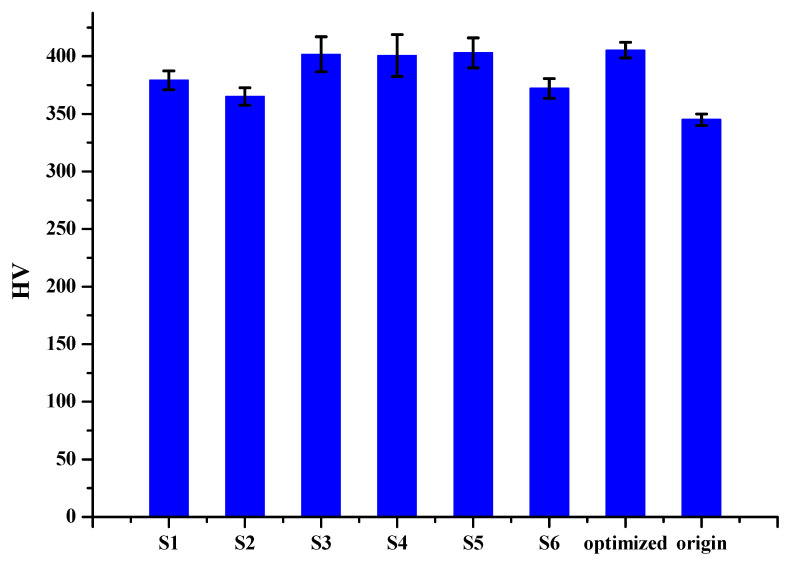
Overall hardness for each sample.

Table 8 shows the annealing data, which has two factors of time and temperature and is differentiated by UDE. After annealing, as shown in Figure 11, samples were taken to the hardness test. The hardness of porous SLM Ti64ELI was measured by the HMV-2 microhardness test machine. The measurement positions were top, middle, and bottom. Each area was taken as three points and obtained at a load of 4.903 N with the duration time of 15 seconds. The result showed that after the annealing process, the hardness of Ti64ELI samples would change obviously, as shown in Table 9. From sample 1 to sample 6, the hardness values were 379.111 HV, 365.111 HV, 401.778 HV, 400.667 HV, 402.889 HV, and 372.111 HV. These were all higher than before the annealing process. The original sample without annealing was about 340~350 HV. After the annealing process, the highest hardness was located in the range of temperature between 530 °C to 610 °C and the annealing time was 40 min to 200 min. Therefore, in this study, the MATLAB CADE was used to find out the best parameter for annealing. The results are shown in Figure 12. The optimal parameter is the annealing temperature 547 °C, and duration was 105 minutes. Then, the annealing process based on the optimal parameter was carried out again to compare with each other, as shown in Table 10 and Figure 13. The optimal value was 409.708 HV and the actual value was 405.333 HV. The actual result was close to the algorithm.

### 3.3. Mechanical Test Results

#### 3.3.1. Compression

**Figure 14:**
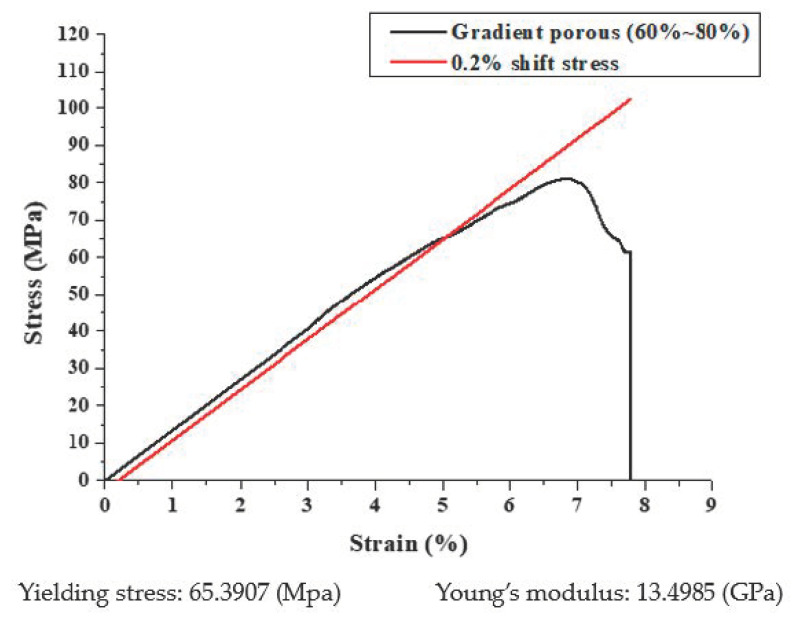
The compressive stress-strain curves of the gradient porosity structure.

According to the previous literature, Young’s modulus and yielding stresses are also listed in Table 11. For porous Ti64ELI scaffolds, as the porosity rose from 43% to 71%, the Young’s modulus decreased from 55.8 to 7.8 GPa and the yield stress decreased from 565 to 62 MPa [43]. The compressive stress-strain curves of the porous Ti64ELI SLM sample is shown in Figure 14. It shows that the Young’s modulus was 13 GPa with the 0.2% shift stress to obtain the yielding stress of 65 MPa. Additionally, the porous Ti64ELI SLM sample with 67% actual porosity, showed a Young’s modulus of 15 GPa and yield stress of 129 MPa, similar to human bone in terms of mechanical properties [44]. The porous Ti64ELI SLM sample with an actual porosity of 67% presented a Young’s modulus and yield stress of about 15 GPa and 129 MPa, respectively, which are similar to the mechanical properties of human bone [44]. Compared with the typical range of Young’s modulus and yield strength of human cortical bone and cancellous bone of 4–30 GPa and 20–193 MPa [23,25], the mechanical properties of the current SLM porous Ti64ELI samples offer highly compatible characteristics that can avoid the risk of the stress shielding effect.

#### 3.3.2. Dynamic Test

**Figure 15:**
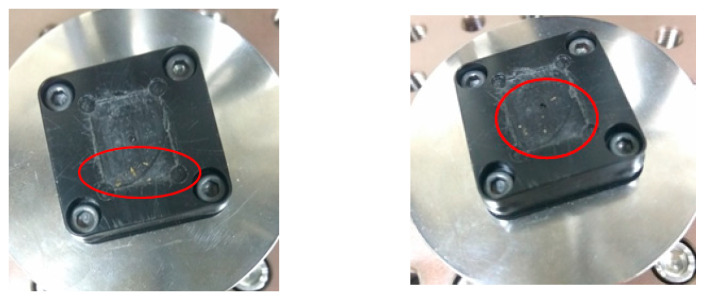
Scraps after the dynamic test.

**Figure 16:**
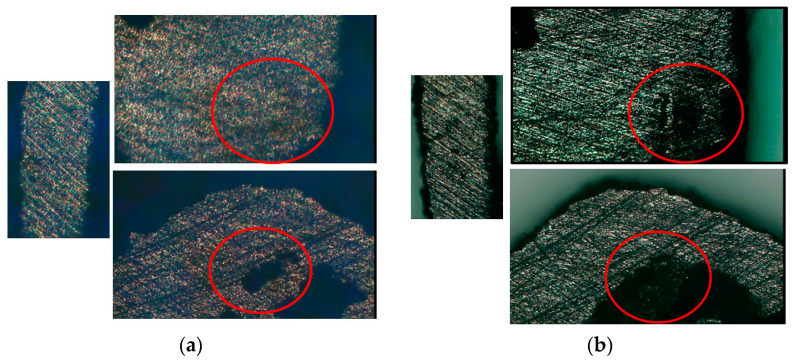
Specimens observed under the OM. (**a**) Before test; (**b**) after test.

There were three steps of the test. Before starting, the measurement points on the specimens were recorded, so that the test pieces could be compared before and after. One of the intuitive features is height. Each specimen recorded the height of corners. The results are shown in Table 12, and there was no obvious change. The other one is surface crack. Each specimen was observed under the OM and images were taken to see if there was any change after the test. The results are shown in Figure 15 and Figure 16 where there were some cracks on the beams. However, there was no obvious change in the exterior, and the cracks were very small, so the cracks might have occurred because of the oxide layer caused by the annealing process.

**Figure 17:**
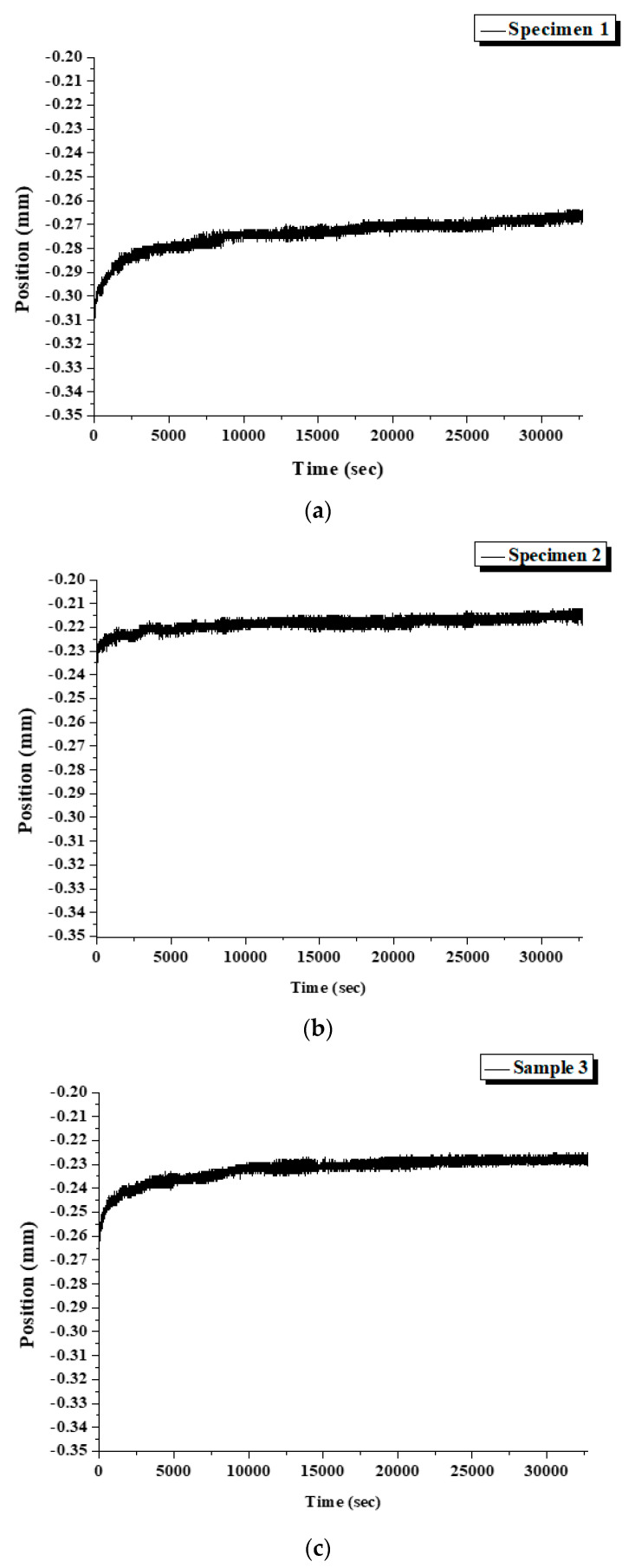
The displacement of specimens during the test. (**a**) Specimen 1, (**b**) Specimen 2, (**c**) Specimen 3.

For the dynamic compression tests, specimens were separated into small pieces to fit the fixture and loaded to 25% of their yield load with 1.5 million times. During the testing to 25% yield load, the displacement was about 0.02 mm without any big changes, as shown in Figure 17. All three specimens survived the test.

### 3.4. Simulation Results

In this simulation, the load was serially applied by Lumbar 1 (L1) on the interbody fusion cage and by the interbody fusion cage on Lumbar 4 (L4). Fusion cages for spinal fusion surgery should not produce any stress-shielding effect. However, if moments are loaded on the vertebrae and intervertebral discs, a mismatch between the fusion cage and the vertebra elastic modulus can cause a non-uniform stress distribution and stress concentration. When the porosity distribution of a fusion cage (i.e., its elastic modulus) matches those of the upper and lower vertebrae, the induced stress and strain on the fusion cage can be reduced. According to Table 13 and Table 14, when the gradient porosity of the cage, from upper to bottom, was 80–70–60–70–80%, the strain of the gradient porosity cage was lower than when the gradient porosity of the cage, from inside to outside, was 60–70–80%. Clearly, the trends of the gradient fusion cages, particularly the maximum stress and strain distributions for these motion modes, showed high agreement with the trends of the simulated intervertebral discs. This demonstrates that the gradient fusion cages exhibit potential applications to future fusion implants. This study designed devices with a high-porosity contact part with the vertebra to demonstrate the ability to design such devices.

**Figure 18:**
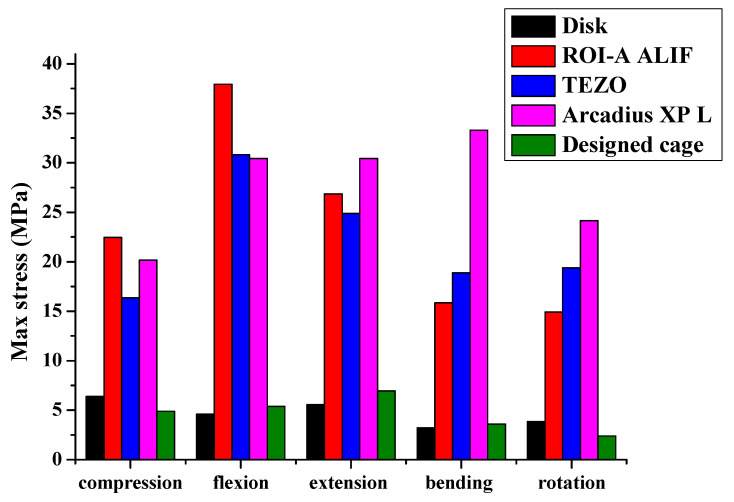
Stress as a function of fusion cages for five different motion modes.

Three commercial cages, ROI-A ALIF (Zimmer, USA), TEZO (Ulrich, Germany), and Arcadius XP L (B. Braun, Germany), were compared with the designed cage and disk, and the stress-strain values are shown in Table 15, Table 16, Table 17, Table 18 and Table 19. The simulated results were compared with those of the intervertebral disc. For all commercial cages, although the devices exhibited lower strain, they had higher stress, leading to stress concentration. These results suggest that fusion cages should be fabricated with gradient porosity for long-term implantation; these devices can trade off the dilemma between stress and strain to avoid the risk of bone fracture over long periods. The simulated results reveal that both the stress and strain behaviors of the fusion cages with gradient porosity showed closer agreement with the intervertebral disc, as demonstrated by the 60–70–80% device shown in Figure 18. This indicates that fusion cages could be optimized in terms of stress and strain and could be customized for each patient’s individual needs and personal bone structure, as seen in Table 15, Table 16, Table 17, Table 18 and Table 19.

## 4. Conclusions

This study presents the gradient porous Ti64ELI structures, intended for application as a replacement for human cortical bone and cancellous bone, which were successfully fabricated by selective laser melting (SLM). The CAD designed structures contain various porosity levels in the range from 60 to 80%, with pore sizes from 600 to 5000 um, suitable for bone tissue in-growth. The SLM structure samples with gradient porosity matched well with their original CAD designs. The difference between the CAD designed and experimentally measured values for both pore size and ligament width was about <15%. The discrepancy between CAD and SLM pore size and ligament width was mainly caused by the laser beam broadening. If we need to improve and produce more precise SLM pores or other morphologies, the powder size and the laser beam size both need to be narrowed down. By doing the UDE and GA method, the optimal annealing parameter is a temperature 547 °C and duration of 105 minutes. The Young’s modulus data on the porous Ti64ELI scaffolds decreased from 110 to 13.5 GPa, and the yield stress data decreased from 990 to 65.39 MPa. The sample with actual porosity presented a Young’s modulus of 13.5 GPa, which matched well with the mechanical properties of human bone, avoiding the risk of the stress shielding effect. For all commercial cages, although the devices exhibited lower strain, they had higher stress, leading to stress concentration. These results suggest that fusion cages should be fabricated with gradient porosity for long-term implantation; these devices can trade off the dilemma between stress and strain to avoid the risk of bone fracture over long periods. Gradient-porosity devices avoided stress concentration. This suggests that gradient-porosity cages might avoid the risk of bone fracture even if they were implanted for long periods. The results also suggest that fusion cages could be optimized in terms of stress and strain and could be customized for the personal needs and distinct bone structures of individual patients.

## Figures and Tables

**Table 1:** The material properties for simulation.

Material	E (GPa)	ν	Density (g/cm^3^)	Yield Strength (GPa)
Cortical bone	12	0.3	1.7	-
Cancellous bone	0.1	0.2	1.1	-
Annulus fibers	0.55	0.3	1	-
Nucleus pulposus	0.001	0.4999	1.02	-
Anterior longitudinal	0.015	0.3	1	-
Posterior longitudinal	0.02	0.3	1	-
Ligamentum flavum	0.019	0.3	1	-
Intertransverse	0.059	0.3	1	-
Capsular	0.033	0.3	1	-
Interspinous	0.012	0.3	1	-
Supraspinous	0.015	0.3	1	-
Ti64ELI	110	0.31	4.43	0.99
Ti64ELI 60% porous	24.4	0.3	1.76	0.23
Ti64ELI 70% porous	9.7	0.3	1.27	0.06
Ti64ELI 80% porous	2.42	0.3	0.66	0.02

**Table 2:** Dimensions of the left part of the cage.

(a) Left (mm)	Avg.	σ	Origin
Width	9.44	0.25	9.5
Height	5.99	0.25	6
Depth	15.93	0.33	16

**Table 3:** Dimensions of the middle part of the cage.

(b) Middle (mm)	Avg.	σ	Origin
Width	11.70	0.20	11.8
Height	6.06	0.03	6
Depth	18.18	0.43	18.4

**Table 4:** Dimensions of the right part of the cage.

(c) Right (mm)	Avg.	σ	Origin
Width	11.20	0.43	11.3
Height	6.08	0.25	6
Depth	16.42	0.33	16.5

**Table 5:** Beam dimensions measured by the scanning electron microscope (SEM) from specimens 1 to 6.

Middle (mm)	Avg.	σ	Origin
A	0.277	0.015	0.25
B	0.229	0.029	0.20
C	0.655	0.059	0.64

**Table 6:** Hole dimensions measured by the SEM form specimens 1 to 6.

Middle (mm)	Avg.	σ	Origin
A	1.063	0.018	1.22
B	3.373	0.113	3.54
C	2.188	0.173	2.34

**Table 7:** Dimensions measured by the optical microscope (OM) from specimens 1 to 6.

Middle (mm)	Avg.	σ	Origin
I	0.2317	0.0280	0.12
II	0.2328	0.0169	0.21
III	0.4926	0.0458	0.59

**Table 8:** The uniform design experimentation (UDE) method for annealing parameters.

Sample	Temperature (°C)	Time (min)
1	450	120
2	490	240
3	530	80
4	570	200
5	610	40
6	650	160

**Table 9:** Overall hardness for each specimen.

Sample	σ	HV
1	2.183	379.111
2	2.055	365.111
3	3.794	401.778
4	4.546	400.667
5	3.247	402.889
6	2.331	372.111
Origin		340~350

**Table 10:** The actual value of the optimal parameter.

	1	2	3	σ	HV
**Top**	404	406	404	0.943	404.667
**Middle**	405	405	406	0.471	405.333
**Bottom**	401	407	410	3.742	406

Total HV: 405.333; σ: 2.309

**Table 11:** Mechanical properties of different porosity for Ti64ELI [43].

Designed Porosity (%)	Real Porosity (%)	Young’s Modulus (GPa)	Yield Stress (MPa)
40	43 ± 0.4	55.0 ± 2.4	564.7 ± 3.1
50	49 ± 0.9	44.4 ± 1.3	465.4 ± 2.1
60	60 ± 0.4	24.4 ± 1.0	233.9 ± 3.4
70	67 ± 0.4	15.3 ± 1.4	128.7 ± 5.6
80	71 ± 0.1	9.7 ± 1.9	62.0 ± 7.9

**Table 12:** Height of each observation point for specimens 1 to 3.

No.	T (mm)	T′ (mm)	R (mm)	R′ (mm)	L (mm)	L′ (mm)
1	4	4	4	4	3.5	3.5
2	4.5	4.5	4	4	3	3
3	4.5	4.5	4	4	3.5	3.5

**Table 13:** The max stress and strain of the horizontally porous gradient structure.

Horizontally Porous	Vertical Compression	Flexion	Extension	Bending	Rotation
Max Stress (MPa)	10.727	18.25	9.847	10.904	13.01
Max Strain (%)	0.045	0.737	0.410	0.454	1.408

**Table 14:** The max stress and strain of the vertically porous gradient structure.

Vertically Porous	Vertical Compression	Flexion	Extension	Bending	Rotation
Max Stress (MPa)	6.324	8.982	7.581	7.614	9.193
Max Strain (%)	0.256	0.372	0.308	0.325	0.988

**Table 15:** The max stress and strain of the ROI-A ALIF model.

ROI-A ALIF	Vertical Compression	Flexion	Extension	Bending	Rotation
Max Stress (MPa)	22.462	37.951	26.875	15.841	14.943
Max Strain (%)	0.0028	0.0051	0.0038	0.0019	0.0015

**Table 16:** The max stress and strain of the TEZO model.

TEZO	Vertical Compression	Flexion	Extension	Bending	Rotation
Max Stress (MPa)	16.349	30.813	24.876	18.899	19.383
Max Strain (%)	0.0016	0.0029	0.0023	0.0018	0.0019

**Table 17:** The max stress and strain of the Arcadius XP L model.

Arcadius XP L	Vertical Compression	Flexion	Extension	Bending	Rotation
Max Stress (MPa)	20.183	30.441	30.429	33.284	24.15
Max Strain (%)	0.0022	0.0031	0.0032	0.0038	0.0023

**Table 18:** The max stress and strain of the designed cage.

Designed Cage	Vertical Compression	Flexion	Extension	Bending	Rotation
Max Stress (MPa)	4.888	5.383	6.942	3.601	2.4
Max Strain (%)	0.202	0.240	0.287	0.149	0.12

**Table 19:** The max stress and strain of the disk.

Disk	Vertical Compression	Flexion	Extension	Bending	Rotation
Max Stress (MPa)	6.398	4.61	5.568	3.246	3.864
Max Strain (%)	0.143	0.405	0.254	0.38	0.07

## Data Availability

Not applicable.
